# Supplementary material for: Modeling and Cost Benefit Analysis to Guide Deployment of POC Diagnostics for Non-typhoidal Salmonella Infections with Antimicrobial Resistance
Source: Sci Rep. 2019 Aug 2;9:11245. doi: 10.1038/s41598-019-47359-2 (PMC6677775; doi:10.1038/s41598-019-47359-2)
Supplement: Supplementary file 1 — Supplemental [file 41598_2019_47359_MOESM1_ESM.pdf]

Modeling and Cost Benefit Analysis to Guide Deployment of POC Diagnostics for Non-typhoidal *Salmonella* Infections with Antimicrobial Resistance

Carrie Manore[1,+,*], Todd Graham[2,+], Alexa Carr[2,+], Alicia Feryn[2], Shailja Jakhar[3], Harshini Mukundan[4], and Hannah Callender Highlander[2]

[1]Los Alamos National Laboratory, Theoretical Biology and Biophysics, Los Alamos,87544, USA
[2]University of Portland, Mathematics Department, Portland, 97203, USA
[3]Los Alamos National Laboratory, Chemistry, Los Alamos, 87544, USA
[4]Los Alamos National Laboratory, Biology, Los Alamos, 87544, USA
[*]cmanore@lanl.gov
[+]these authors contributed equally to this work

ABSTRACT

Invasive non-typhoidal *Salmonella* (NTS) is among the leading causes of blood stream infections in sub-Saharan Africa and other developing regions, especially among pediatric populations. Invasive NTS can be difficult to treat and have high case-fatality rates, in part due to emergence of strains resistant to broad-spectrum antibiotics. Furthermore, improper treatment contributes to increased antibiotic resistance and death. Point of care (POC) diagnostic tests that rapidly identify invasive NTS infection, and differentiate between resistant and non-resistant strains, may greatly improve patient outcomes and decrease resistance at the community level. Here we present for the first time a model for NTS dynamics in high risk populations that can analyze the potential advantages and disadvantages of four strategies involving POC diagnostic deployment, and the resulting impact on antimicrobial treatment for patients. Our analysis strongly supports the use of POC diagnostics coupled with targeted antibiotic use for patients upon arrival in the clinic for optimal patient and public health outcomes. We show that even the use of imperfect POC diagnostics can significantly reduce total costs and number of deaths, provided that the diagnostic gives results quickly enough that patients are likely to return or stay to receive targeted treatment.

Supplemental Material

Supplementary Material 1: Equations for Full Model Note below that the κ^0 notation means diagnostic time is NOT added to the total treatment time since diagnostics are not applied, i.e. $\phi = 0$. Scenario 2 equations:

$$\begin{aligned}\frac{dS}{dt} &= -\alpha S(I_s + J_s + I_m + J_m) + \kappa^0_{ar} I_s + \kappa^0_a J_s + \rho[\sigma\kappa_r I_m + (1-\sigma)\kappa_{ar} I_m + \\ &\lambda\kappa_a J_m + (1-\lambda)\kappa_{ra} J_m] + \theta(I_m + J_m) - \alpha_w S(W_I + W_J)\end{aligned} \tag{1}$$

$$\frac{dI_m}{dt} = \alpha(1-\beta)S(I_s + I_m) - \rho[\sigma\kappa_r I_m + (1-\sigma)\kappa_{ar} I_m] - \mu_m I_m - \theta I_m + \alpha_w W_I S(1-\beta) \tag{2}$$

$$\frac{dJ_m}{dt} = \alpha(1-\gamma)S(J_s + J_m) - \rho[\lambda\kappa_a J_m + (1-\lambda)\kappa_{ra} J_m] - \mu_m J_m - \theta J_m + \alpha_w W_J S(1-\gamma) \tag{3}$$

$$\frac{dI_s}{dt} = \alpha\beta S(I_s + I_m) - \kappa^0_{ar} I_s - \mu_s I_s + \alpha_w W_I S\beta \tag{4}$$

$$\frac{dJ_s}{dt} = \alpha\gamma S(J_s + J_m) - \kappa^0_a J_s - \mu_s J_s + \alpha_w W_J S\gamma \tag{5}$$

$$\frac{dR}{dt} = \mu_s(I_s + J_s) + \mu_m(I_m + J_m) \tag{6}$$

$$\frac{dW_I}{dt} = \tau(I_s + I_m) - W_I\theta \tag{7}$$

$$\frac{dW_J}{dt} = \tau(J_s + J_m) - W_J\theta \tag{8}$$

Scenario 3 equations:

$$\frac{dS}{dt} = -\alpha S(I_s+J_s+I_m+J_m)+\kappa_{ar}^0(I_s+I_m)+\kappa_a^0(J_s+J_m)+\theta(I_m+J_m)-\alpha_w S(W_I+W_J) \tag{9}$$
$$\frac{dI_m}{dt} = \alpha(1-\beta)S(I_s+I_m)-\kappa_{ar}^0 I_m-\mu_m I_m-\theta I_m+\alpha_w W_I S(1-\beta) \tag{10}$$
$$\frac{dJ_m}{dt} = \alpha(1-\gamma)S(J_s+J_m)-\kappa_a^0 J_m-\mu_m J_m-\theta J_m+\alpha_w W_J S(1-\gamma) \tag{11}$$
$$\frac{dI_s}{dt} = \alpha\beta S(I_s+I_m)-\kappa_{ar}^0 I_s-\mu_s I_s+\alpha_w W_I S\beta \tag{12}$$
$$\frac{dJ_s}{dt} = \alpha\gamma S(J_s+J_m)-\kappa_a^0 J_s-\mu_s J_s+\alpha_w W_J S\gamma \tag{13}$$
$$\frac{dR}{dt} = \mu_s(I_s+J_s)+\mu_m(I_m+J_m) \tag{14}$$
$$\frac{dW_I}{dt} = \tau(I_s+I_m)-W_I\theta \tag{15}$$
$$\frac{dW_J}{dt} = \tau(J_s+J_m)-W_J\theta \tag{16}$$

Scenario 4 equations:

$$\frac{dS}{dt} = -\alpha S(I_s+J_s+I_m+J_m)+\kappa_{ar}^0 I_s+\kappa_a^0 J_s+\theta(I_m+J_m)-\alpha_w S(W_I+W_J) \tag{17}$$
$$\frac{dI_m}{dt} = \alpha(1-\beta)S(I_s+I_m)-\mu_m I_m-\theta I_m+\alpha_w W_I S(1-\beta) \tag{18}$$
$$\frac{dJ_m}{dt} = \alpha(1-\gamma)S(J_s+J_m)-\mu_m J_m-\theta J_m+\alpha_w W_J S(1-\gamma) \tag{19}$$
$$\frac{dI_s}{dt} = \alpha\beta S(I_s+I_m)-\kappa_{ar}^0 I_s-\mu_s I_s+\alpha_w W_I S\beta \tag{20}$$
$$\frac{dJ_s}{dt} = \alpha\gamma S(J_s+J_m)-\kappa_a^0 J_s-\mu_s J_s+\alpha_w W_J S\gamma \tag{21}$$
$$\frac{dR}{dt} = \mu_s(I_s+J_s)+\mu_m(I_m+J_m) \tag{22}$$
$$\frac{dW_I}{dt} = \tau(I_s+I_m)-W_I\theta \tag{23}$$
$$\frac{dW_J}{dt} = \tau(J_s+J_m)-W_J\theta \tag{24}$$

Supplementary Material 2: Basic Reproduction Number for Simple Model (no Env compartment)

For Scenario 2, the basic reproduction number for the non-resistant strain is

$$\mathscr{R}_0^{J,2} = \frac{\alpha\gamma N}{\kappa_a^0+\mu_s}+\frac{\alpha(1-\gamma)N}{\theta+\mu_m+\rho(\lambda\kappa_a+(1-\lambda)\kappa_{ra})}$$

and for the resistant strain is

$$\mathscr{R}_0^{I,2} = \frac{\alpha\beta N}{\kappa_{ar}^0+\mu_s}+\frac{\alpha(1-\beta)N}{\theta+\mu_m+\rho(\sigma\kappa_r+(1-\sigma)\kappa_{ar})}.$$

For Scenario 3, the basic reproduction number for the non-resistant strain is

$$\mathscr{R}_0^{J,3} = \frac{\alpha\gamma N}{\kappa_a^0+\mu_s}+\frac{\alpha(1-\gamma)N}{\theta+\mu_m+\kappa_a^0}$$

and for the resistant strain is

$$\mathscr{R}_0^{I,3} = \frac{\alpha\beta N}{\kappa_{ar}^0+\mu_s}+\frac{\alpha(1-\beta)N}{\theta+\mu_m+\kappa_{ar}^0}.$$

Supplementary Material For Environmental/Outside Compartment Model

Table 1. Costs of diagnostic deployment and antibiotic use for each scenario with $\alpha_w = 0.5 * \alpha$ where A is the cost of standard antibiotic treatment (effective on sensitive strain), R the cost of resistant strain treatment, and D the cost of the diagnostics. All costs are in U.S. Dollars (USD).

Scenario	Diagnostic Used	Cost A (USD)	Cost AR (USD)	Cost DA (USD)	Cost DAR (USD)	Cost DR (USD)	Total Cost (USD)
1	Antibody	0	0	115,448	59,618	422,811	597,877
	BC	0	0	348,864	182,898	492,758	1,024,519
	PCR	0	0	509,918	72,421	503,883	1,086,221
2	Antibody	44,339	1,114,363	15,007	30,434	647,765	1,851,908
	BC	44,087	1,235,844	13,165	35,852	440,452	1,769,400
	PCR	44,480	1,218,513	19,371	14,732	548,027	1,845,124
3	None	51,958	1,665,318	0	0	0	1,717,277
4	None	44,567	1,397,902	0	0	0	1,442,468

Table 2. Number of deaths from NTS, percent of cases improperly treated, and the number of diagnostics used in each scenario with $\alpha_w = 0.5 * \alpha$ run for 1,000 days. $\rho = 0.6$ for BC and PCR.

Scenario	Diagnostic Used	Number Deaths (People)	Improperly Treated (Percent)	Num. Diagnostics Deployed
1	Antibody	4,031	4.2%	19,796
	BC	14,756	6.7%	35,784
	PCR	14,115	2.5%	35,589
2	Antibody	11,708	51.6%	12,224
	BC	12,919	61.3%	7,982
	PCR	12,759	58.6%	8,795
3	None	13,268	81.1%	None
4	None	14,622	80.8%	None

Table 3. PRCC values, first- and total-order indices with their p-values for measuring the sensitivity of Scenario 1, 2, 3 and 4's non-environmental parameters to model R. Parameters were allowed to vary ±50% of their nominal values. The sample space was obtained using Latin Hypercube sampling. Values with a * have a p value less than 0.05. Recall that κ_*^0 is the treatment/recovery rate when no diagnostic is used.

Scenario	Test	κ_{ar}^0	κ_a^0	κ_{ar}	κ_a	κ_r	μ_s	μ_m	σ_{anti}	λ_{anti}	α	β	γ	θ
1	PRCC	-	-	-0.1309*	-0.7124*	-0.4163*	-0.0562	0.1102*	-0.2127*	-0.3795*	0.8643*	0.3319*	0.6461*	-0.3615*
	S_i	-	-	0.0030*	0.1003*	0.0136*	0.0018*	0.0001	0.0039*	0.0146*	0.3093*	0.0123*	0.0811*	0.0095*
	S_{ti}	-	-	0.0142*	0.1642*	0.0390*	0.0122*	0.0028	0.0166*	0.0374*	0.4026*	0.0408*	0.1417*	0.0189*
2	PRCC	-0.7446*	-0.3471*	-0.0013	0.0252	-0.0893*	-0.3551*	0.0192	-0.0472	0.0473	0.9078*	0.8081*	0.2898*	-0.3504
	S_i	0.1445*	0.0169*	0.0000*	0.0000*	0.0008*	0.0145*	0.0000*	0.0004*	0.0000*	0.4877*	0.2000*	0.0096*	0.0075*
	S_{ti}	0.1902*	0.0505*	0.0002	0.0004*	0.0021*	0.0280*	0.0001	0.0009	0.0002*	0.5347*	0.2589**	0.0331*	0.0123*
3	PRCC	-0.7856*	-0.3280*	-	-	-	-0.2978*	0.0263	-	-	0.9188*	0.7898*	0.2269*	-0.4181*
	S_i	0.1527*	0.0139*	-	-	-	0.0133*	0.0000*	-	-	0.5098*	0.1556*	0.0068*	0.0159*
	S_{ti}	0.1963*	0.0405*	-	-	-	0.0246*	0.0002	-	-	0.5496*	0.2004*	0.0257*	0.0229*
4	PRCC	-0.7550*	-0.3057*	-	-	-	-0.2755*	0.0512	-	-	0.9216*	0.7531*	0.2531*	-0.4931*
	S_i	0.1303*	0.0124*	-	-	-	0.0113*	0.0000*	-	-	0.5430*	0.1357*	0.0055*	0.0356*
	S_{ti}	0.1661*	0.0364*	-	-	-	0.0219*	0.0002*	-	-	0.5770*	0.1746*	0.0213*	0.0484*

Table 4. PRCC values, first- and total-order indices with their p-values for measuring the sensitivity of Scenario 1, 2, 3 and 4's environmental parameters to model R. Parameters were allowed to vary ±50% of their nominal values. The sample space was obtained using Latin Hypercube sampling. Values with a * have a p value less than 0.05.

Scenario	Test	α_w	ψ	τ	θ_w
1	PRCC	0.4952*	0.4340*	0.0905*	-0.4960
	S_i	0.0142*	0.0062*	0.0017*	0.0172*
	S_{ti}	0.0207*	0.0113*	0.0057*	0.0242*
2	PRCC	0.3928*	0.2483*	0.1337*	-0.4131*
	S_i	0.0150*	0.0064*	0.0025*	0.0191*
	S_{ti}	0.191*	0.0084*	0.0036*	0.0231*
3	PRCC	0.4073*	0.2640*	0.1951*	-0.4712*
	S_i	0.0170*	0.0059*	0.0025*	0.0205*
	S_{ti}	0.0217*	0.0078*	0.0036*	0.0249*
4	PRCC	0.3375*	0.2165*	0.1912*	-0.4253*
	S_i	0.0181*	0.0059*	0.0028*	0.0212*
	S_{ti}	0.0232*	0.0078*	0.0039*	0.0257*

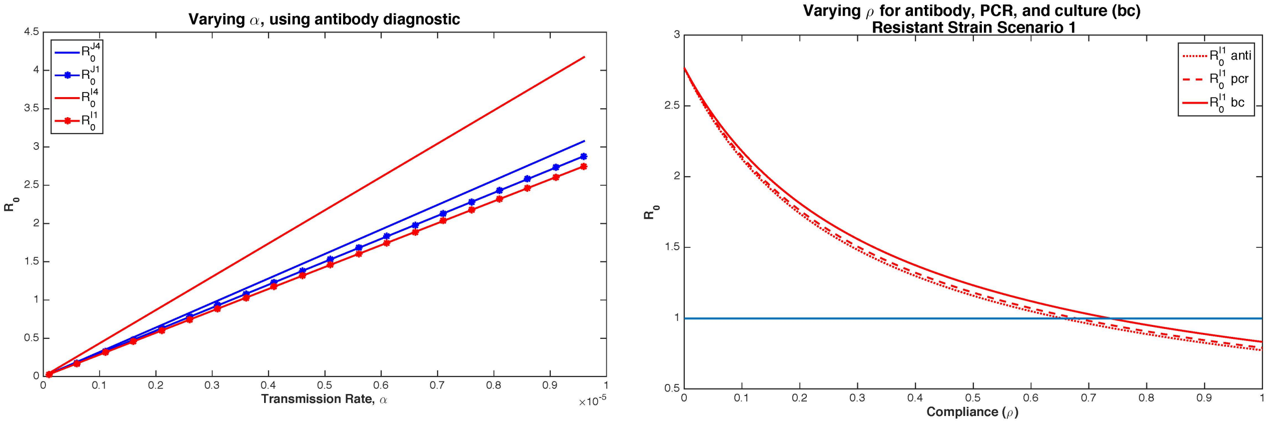

(a) **(b)**

Figure 1. Sensitivity of R_0 components to the transmission rate, α (Figure 1a), for different diagnostics and Scenarios 1 and 4, and to the compliance rate, ρ (Figure 1b). In subfigure 1a, blue lines are the sensitive strain and red the resistant strain. The flat lines are in the absence of diagnostics (Scenario 4) and starred lines with full diagnostic deployment (Scenario 1). In subfigure 1b, the compliance rate is the proportion of people who return to the clinic to receive diagnostic results and an appropriate treatment based on those results. $\mathscr{R}_0$ increases linearly with the transmission rate and decreases non-linearly with compliance. The sensitivity of $\mathscr{R}_0$ on compliance does not depend on diagnostic type.

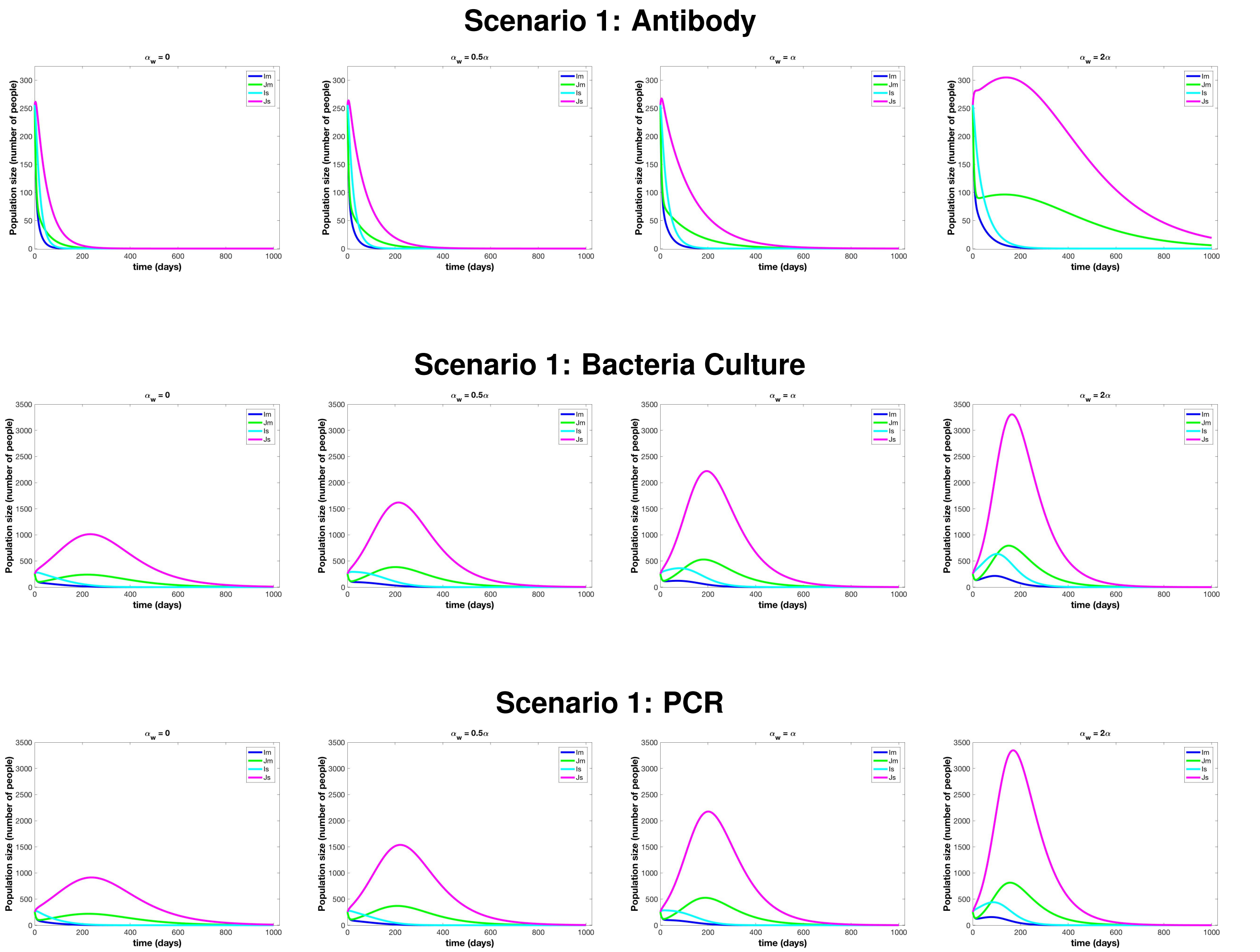

Figure 2. Outcome change with the environmental/low-risk compartment as the outside transmission rate, α_w, changes for Scenario 1. While magnitude changes the general patterns remain the same except for very high values of outside transmission.

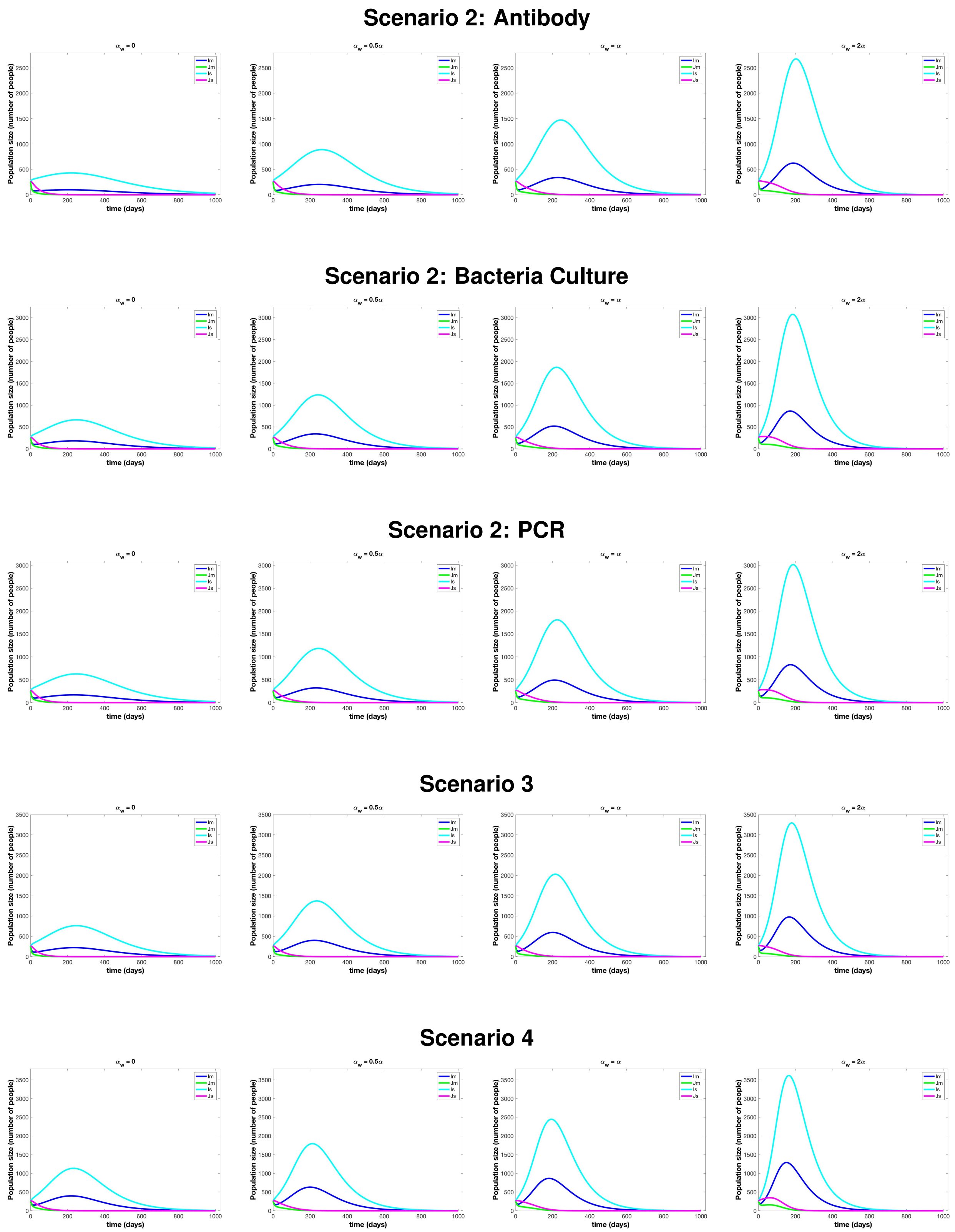

Figure 3. Outcome change with the environmental/low-risk compartment as the outside transmission rate, α_w, changes for Scenarios 2 - 4. While magnitude changes the general patterns remain the same except for very high values of outside transmission.

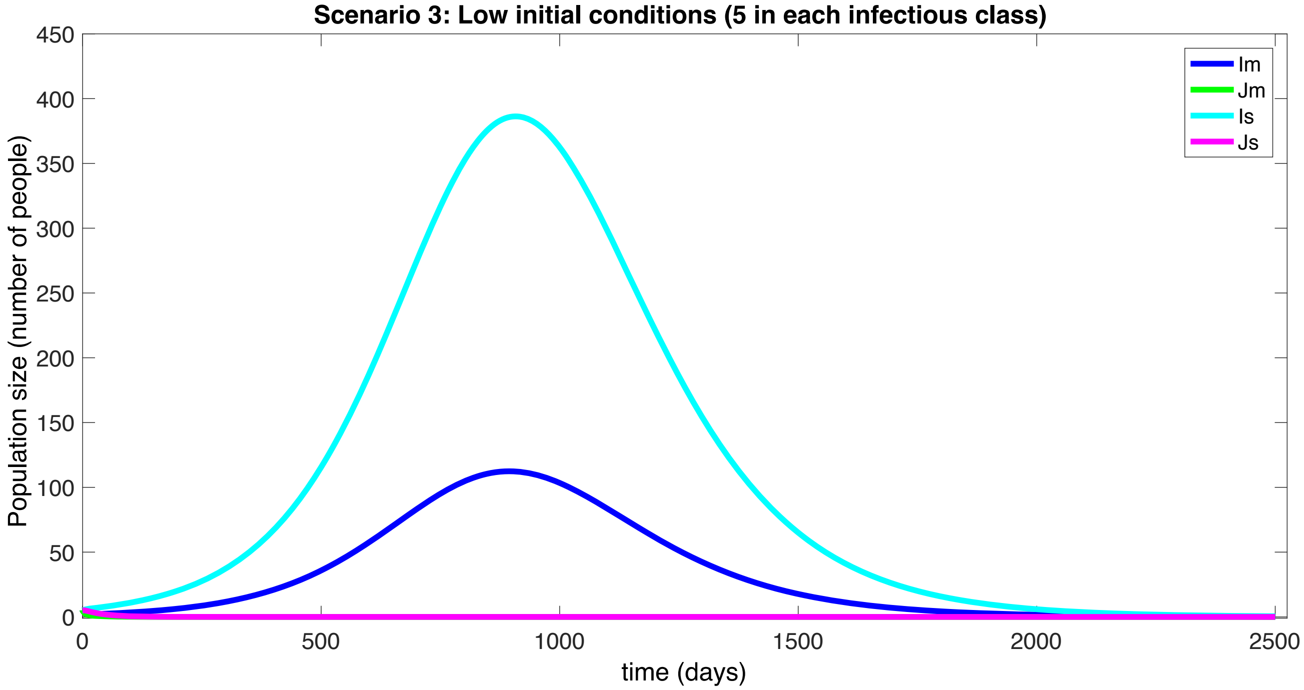

Figure 4. Scenario 3 for low initial conditions to simulate the outbreak in Blantyre, Malawi.
